# Trauma research 2012

**DOI:** 10.1186/1757-7241-21-S1-A10

**Published:** 2013-05-28

**Authors:** AS Johnsen, M Rehn

**Affiliations:** 1 Department of Research, Norwegian Air Ambulance Foundation, Drøbak, Norway; 2 Department of Anaesthesia and Intensive Care, Oslo University Hospital, Oslo, Norway; 3 Department of Anaesthesia and Intensive Care, Akershus University Hospital, Akershus, Norway

## 

Although trauma remains a significant contributor to the global burden of disease, trauma research remains uncoordinated and often of low quality. Research efforts aiming to improve trauma care are drastically underfunded and global research strategies to address core challenges are missing [1,2]. The responsibility for this state lies partially within the trauma academic community and efforts to coordinate research programmes are called for. It was therefore very welcome that Professor Karim Brohi chaired a session dedicated to trauma research on the final day of the London trauma conference 2012. The session included speakers describing activity organised by UK research centres. A common theme was how the establishment of trauma networks offers new research opportunities [3].

The trauma system has the potential to facilitate robust epidemiological and public health research necessary for evidence-based system revisions. Further, formal trauma networks bridge the gap between clinical and basic science research. Pending clearly defined relationships, the trauma networks also facilitate cooperation with the medical industry to make quality improvement initiatives more effective. London is the worlds largest single trauma system and it provides opportunities for robust data collection from the scene of injury throughout rehabilitation. Such data may facilitate long-awaited high quality observational studies and randomized controlled trials on both primary and secondary preventive measures.

Contemporary trauma research is heavily influenced by the current conflict in Afghanistan. Increased military funding has enabled studies to be performed on many aspects of trauma care from pre-hospital interventions to rehabilitation several of which were described in this session.

Although the populations differ with respect to age, gender and co-morbidities, some data collected from the battlefield are considered transferable to the civilian community. A feasibility study of using rotational thromboelastometry to assess coagulation status of combat casualties in a deployed setting provides an example of how military research brings science to the front line [4]. Further, the high number of soldiers returning with lost limbs has initiated research programs into reconstructive challenges. As Western involvement in the Afghanistan conflict draws to an end, the destiny of military funded trauma research remains uncertain.

Civilian and military clinicians share interests in unresolved challenges pertaining to areas such as nerve tissue damage and coagulation. Hypothesis on whether minor head injury can prime inflammation in the brain that may lead to neurodegenerative diseases are currently being investigated. Further, studies on neuroprotective agents for brain and spinal cord injury aims to reduce the morbidity that traumatic brain injury carries. Neuroprotection is also an essential component to improve outcome after peripheral injury. Ongoing studies of reconstruction of nerve conduction through pharmacological intervention shows promising results [5]. Lastly, haemorrhage is a major cause of trauma deaths and translational research on coagulopathy, transfusion and pro-coagulant therapies is currently conducted on several centres.

High-achieving research groups rely on not only support from within their own institution but, interdisciplinary relations as well as national and international networks. These interfaces serve to improve quality and recruit financial and human resources. Research decision-makers are required to map out the need to establish new centres or where it is more cost-effective to tap into existing research clusters. Regardless, the importance of networks was exemplified in trials such as the DRAFFT-study on distal radius fractures, where inclusion rates where good [6]. Lessons can also be learned from areas such as critical care where several randomised controlled trials on core topics such as sepsis have been conducted. However, research on time-critical emergencies remains a challenge as they deviate from planned and organized environments.

High-quality data is a prerequisite to conduct high-quality trials. Optimal data collection involves automatic linkage between databases and bulk uploads to registries such as The UK Trauma Audit and Research Network (TARN). Areas for improvements in data collection include minimizing manual data collection and enabling research nurses to attend trauma meetings. Cancer registries serve as role models with their long tradition for providing reliable and valid data.

The day ended with updates on large, on-going trials such as CRASH 3 [7] and CRYOSTAT [8]. Hopefully, robust international trauma networks will, in the future, attract investment funding in trauma research. Our patients deserve trials with rapid recruitment timelines with greater capacity and diversity to make an impact on clinical practice and improve outcome.
